# Influence of pallet height on energy consumption and cooling effectiveness in an apple cold storage

**DOI:** 10.1038/s41598-025-95886-y

**Published:** 2025-04-04

**Authors:** Leo Daniel Alexander, Sanjeev Jakhar, Mani Sankar Dasgupta

**Affiliations:** 1 Smart Building Laboratory, Department of Mechanical Engineering, Birla Institute of Technology & Science, Pilani, Rajasthan, 333031 India; 2 School of Mechanical Engineering, Vellore Institute of Technology, Chennai, 600127 Tamil Nadu India

**Keywords:** Computational fluid dynamics, Cold storage, Porous medium, Post harvest, Thermal heterogeneity, Engineering, Mechanical engineering

## Abstract

This study investigates the influence of pallet height on energy consumption and cooling effectiveness using a validated cold storage model based on CFD simulations. The model was validated against experimental temperature data, with a maximum normalized root mean square error (NRMSE) of 4.57%, indicating good agreement. Four pallet height configurations namely No pallet (0.0 m), 0.3 m, 0.6 m, and 0.9 m were assessed over a 40-hour cooling period. Temperature distribution within apple-filled crates was used to identify the locations of hot and cold spots, and performance metrics such as compressor energy consumption, specific energy consumption, mean crate temperature, thermal heterogeneity, and cooling effectiveness were analyzed. The results indicate that the No-pallet configuration disrupts airflow resulting in insufficient cooling, as evidenced by lower cooling effectiveness, while larger pallet heights (0.6 m and 0.9 m) introduce excessive air spacing resulting in higher thermal heterogeneity. The 0.3 m pallet height exhibited best performance such as lowest mean crate temperature of 4.7 °C, (8.7% lower), lowest thermal heterogeneity of 3.29 (14.42% lower), lowest compressor energy consumption per kelvin of 1.474 kWh/K (0.5% lower) and highest cooling effectiveness of 0.4 (5.37% higher). These findings provide practical insights for optimizing pallet configurations in cold storage, aiding energy-efficient operations in commercial refrigerated warehouses and post-harvest supply chains.

**Supplementary information:**

The online version contains supplementary material available at 10.1038/s41598-025-95886-y.

## Introduction

Mitigating post-harvest losses is of paramount importance for India. Given the country’s status as an agrarian powerhouse, agriculture contributes to 19.9% of the National GDP^1^. A study estimated that fruits and vegetables contribute 37.31% of the total monetary value in post-harvest loss. Post-harvest losses refer to the reduction in weight and quality of agricultural produce that occurs between the time of harvest and the point of consumption^2^. Apart from the fragmented post-harvest supply chain problem, the diverse weather conditions in India can exacerbate post-harvest losses through various mechanisms such as high temperature and humidity, excessive moisture, physical damage due to faulty handling, pests and insects, etc^3^. , . Cold storage plays a pivotal role in mitigating post-harvest losses in fruits and vegetables, offering a multitude of benefits that contribute to the preservation of quality, extension of shelf life, and reduction of economic losses^4^.

For long-term preservation of horticultural products in cold storage, it is essential to maintain ideal storage conditions. Apples are a fruit of high nutritional value and are harvested seasonally yet maintain a year-round demand^5^. A stochastic model by Gwanpua et al., (2013) revealed that increasing storage temperatures from 1 °C to 3 °C over a 6-month period led to a 15% reduction in apple quality^6^. Another significant challenge in post-harvest storage is gradual loss of moisture, which not only reduces the fruit’s weight and quality but also shortens its shelf-life and lowers profits^7^. Moreover, moisture loss is considered to be a key quality indicator for consumers when purchasing fruit^8^. Research has shown that fruits and vegetables losing more than 5% of their water content become unmarketable due to visible shriveling^9^. In addition, managing uniform temperature and relative humidity within the cold storage is challenging but is a prime requirement in optimizing storage conditions. Gruyters et al., (2018) reported numerical modeling that studies the influence of thermal heterogeneity on apples stored long-term and found that better temperature uniformity within the cold storage can extend the storage duration of apples by up to 14 days^10^.

Employing Computational Fluid Dynamics (CFD) for simulation of the airflow and temperature distribution within a cold storage setup proves to be immensely valuable in the food industry^11^. Alexander et al., (2024) reported CFD simulations of apple storage bins aimed at optimizing airflow distribution within and around the bins^12^. Similarly, J.-W. Han et al., (2019) utilized CFD to identify the optimal turbulence model, a critical factor in fluid flow simulations for cold storage environments^13^. They concluded that the SST (Shear Stress Transport) k-ω model provides more accurate results compared to other models such as the standard k-ε, RNG (Re-Normalization Group) k-ε, Realizable k-ε, and RSM (Reynolds Stress Model). In a later study, J. Han et al., (2021) combined experimental research with CFD analysis to assess how intermittent warming impacts cooling rates, energy efficiency, moisture loss, and chilling injuries in horticultural products stored in cold environments^14^. Yilmaz & Yilmaz, (2020) employed CFD to determine the optimal cold storage capacity and developed a correlation between payback period and storage capacity using historical data for better decision-making in storage facility design^15^. Park et al., (2023) utilized CFD and devised an advanced temperature control algorithm leveraging upon deep reinforcement learning techniques to strategically enhance energy efficiency within operational cold storage facilities and reported a notable accomplishment with a 47.64% reduction in energy consumption^16^. Gou et al., (2023) reported a CFD analysis to study the impact of inlet air velocity and spacing between cold thermal plates on the heat absorption capability of the cold storage plates^17^.

Among the various factors that influences the rate of cooling inside a cold storage, airflow distribution is of prime importance^11^. This is due to the fact that, air flow distribution has the potential to enhance the overall performance of cold storage facility, curtail operational expenses and establish a uniform temperature distribution in the cold storage without increasing the capital investment^18^. The dynamics of air circulation within a cold storage setting is significantly affected by the available void space for the passage of chilled air. The magnitude of this void space is intricately linked to the dimensions and configuration of the pallets and crates instrumental in housing the stored products. Tiamiyu et al., (2022) demonstrated an application of CFD in a comprehensive analysis of the effect of void plugs used to cover the unutilized floor area between the cold storage door and pallet stacks^19^. Liu et al., (2019) proposed a novel pallet design incorporating water as a thermal energy storage medium in a refrigerated storage truck^20^. Liu et al., (2021) also proposed a novel PCM (Phase Change Materials) based pallet used in refrigerated storage trucks for better temperature control performance in the cold chain^21^. Han et al., (2018) utilized CFD to evaluate the parameters such as cooling rate, temperature uniformity, moisture loss and energy utilization during precooling in forced air cooling of apple crates stacked over pallets^22^.

Previous literature has emphasized the importance of spacing between crates and the use of retrofitted fans and ducts to enhance airflow distribution inside cold storage, thereby improving cooling effectiveness. However, the impact of pallet height in overall air flow distribution has received little attention. This research aims to address this gap. The objective of this research is to present a comprehensive CFD analysis of the influence of pallet height on the temperature of the products stored inside the cold storage to find an optimum pallet height at which the cooling rate will be most favorable. By improving cooling efficiency and reducing post-harvest losses, this study supports food security and availability, aligning with Sustainable Development Goal 2 (SDG 2): Zero Hunger^23^. Optimizing pallet height also enhances energy efficiency, reducing wastage and promoting sustainable storage practices, contributing to SDG 12: Responsible Consumption and Production^24^. Furthermore, more efficient and accessible cold storage solutions benefit marginal farmers by minimizing spoilage, ensuring just price for their produce addressing SDG 1: No Poverty^25^.

## Materials and methods

### System description

**Fig. 1:**
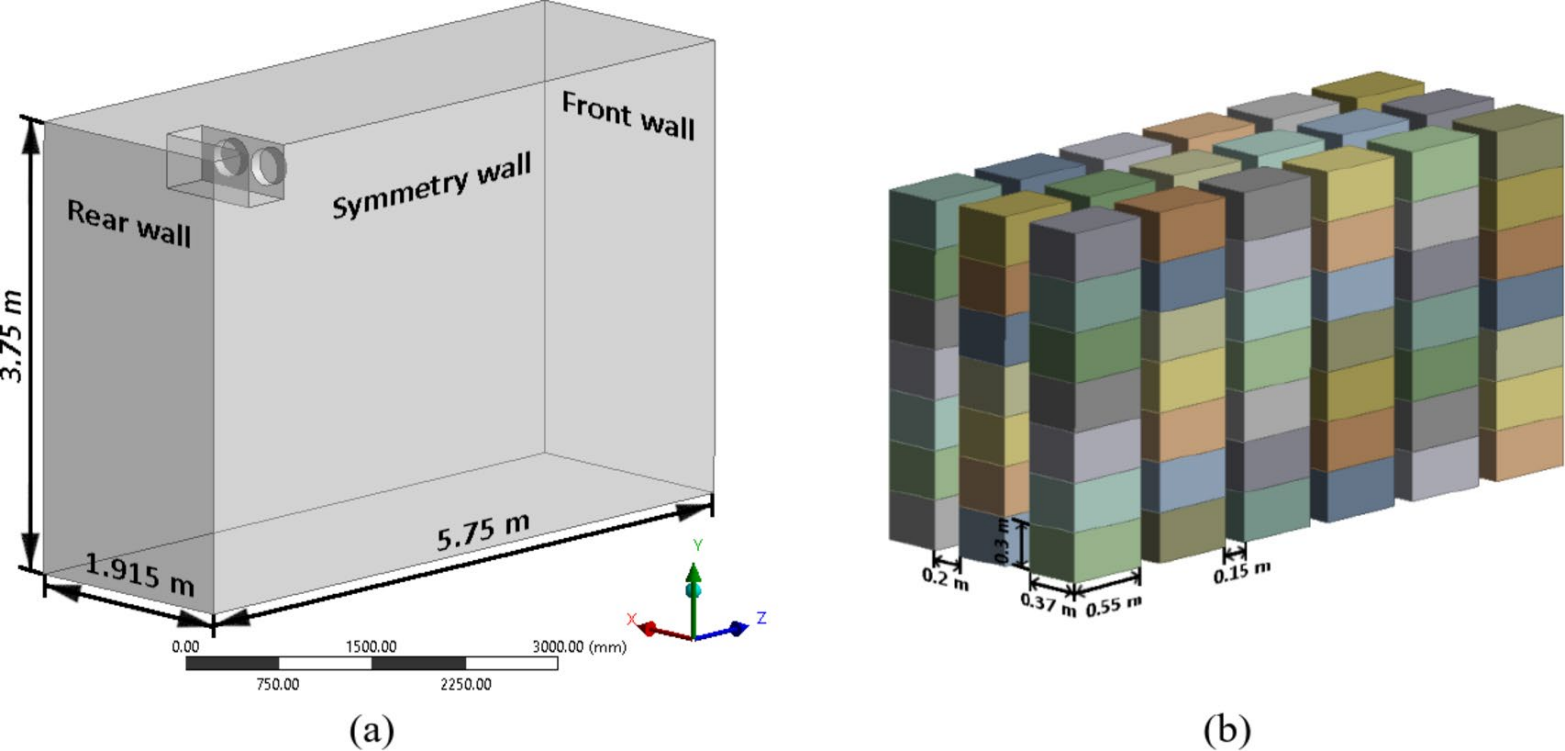
(**a**) Cold storage geometry, (**b**) Apple crate dimensions and spacing.

**Fig. 2:**
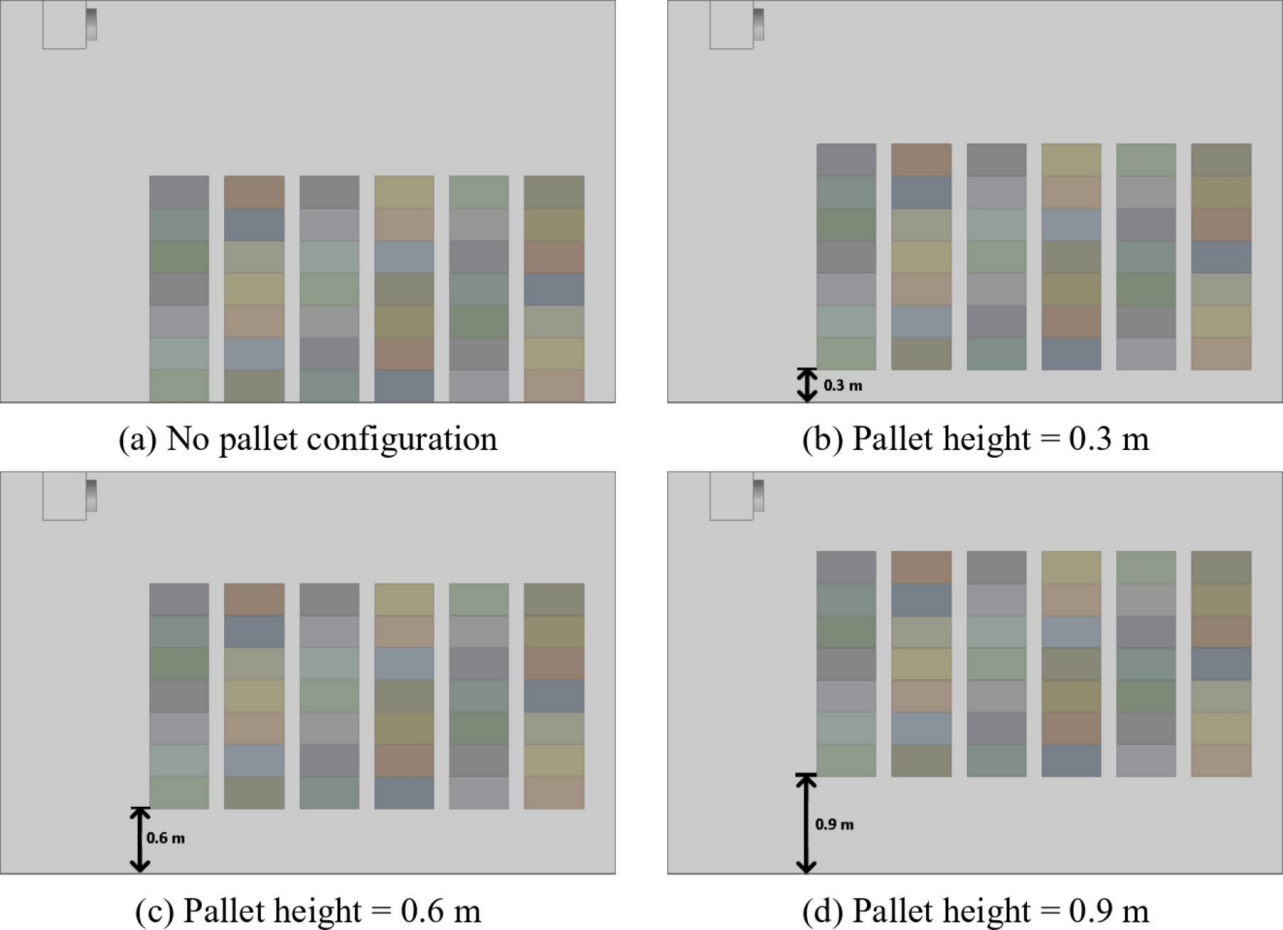
(**a**) No pallet configuration, (**b**) Pallet height = 0.3 m, (**c**) Pallet height = 0.6 m, (**d**) Pallet height = 0.9 m.

The cold storage identified to develop the numerical modelled is as per the reported experimental study by Bishnoi & Aharwal, (2020)^26^. The geometry of the modelled cold storage is shown in Fig. 1 (a) which comprises of dimensions 5.75 m × 3.75 m × 1.915 m. It is imperative to note that, owing to the inherent symmetry of the cold storage and the strategic arrangement of crates, a symmetry boundary condition is assumed. Consequently, the modelling of only half of the cold storage is undertaken, which reduces the computational burden and enhances efficiency of the analysis. The crates are of standard dimension of 0.55 m × 0.37 m × 0.3 m and are stacked on top of each other in continuity. The stacked crates are systematically arranged on both sides featuring a spacing of 0.15 m along the Z direction and 0.2 m along the X direction as shown in Fig. 1(b). A total of 126 crates were placed and these were incorporated into the cold storage geometry. As per the objective of the paper, to identify the influence of pallet height on the crate temperature, four pallet configurations (Fig. 2a, b, c and d) of heights 0 m, 0.3 m, 0.6 m, and 0.9 m are modelled. Four pallet height configurations (0 to 0.9 m, in 0.3 m increments) were selected. Heights above 0.9 m were avoided as they could obstruct airflow to crates near the front wall. Intermediate values were excluded due to minimal expected variation in airflow distribution. To ensure that the model can be universally applied, the physical pallets are not modelled but only the air gap created by them is considered, it is assumed that the heat capacity of pallets themselves are negligible compared to the apple filled crates and their effect on airflow distribution within the cold storage is negligible. The CFD model reduces experimental costs but assumes idealized boundaries, and simplified turbulence, which may cause deviations from real cold storage operations with dynamic ambient and complicated turbulence.

### Governing equations

The fluid flow inside the cold storage is governed by the continuity and momentum equation as shown in Eqs. (1) and (2).

1 $$\:\frac{\partial\:{\rho\:}_{a}}{\partial\:t}+\stackrel{-}{\nabla\:}\left({\rho\:}_{a}\stackrel{-}{u}\right)=0$$

2 $$\:\frac{\partial\:{\rho\:}_{a}{\stackrel{-}{u}}_{i}}{\partial\:t}+\frac{\partial\:{\rho\:}_{a}{\stackrel{-}{u}}_{i}{\stackrel{-}{u}}_{j}}{\partial\:{x}_{j}}=-\frac{\partial\:\stackrel{-}{p}}{\partial\:{x}_{i}}+\frac{\partial\:}{\partial\:{x}_{j}}\left[\mu\:\left(\frac{\partial\:{\stackrel{-}{u}}_{i}}{\partial\:{x}_{j}}+\frac{\partial\:{\stackrel{-}{u}}_{j}}{\partial\:{x}_{i}}\right)\right]-\frac{\partial\:{\rho\:}_{a}\stackrel{-}{{u}_{i}^{{\prime\:}}{u}_{j}^{{\prime\:}}}}{\partial\:{x}_{j}}+{S}_{i}$$

where $\:{\rho\:}_{a}$ represents fluid density $\:\left(kg/{m}^{3}\right)$, $\:{\stackrel{-}{u}}_{i}$ and $\:{\stackrel{-}{u}}_{j}$ represents the mean velocity component in $\:i$ and $\:j$ direction $\:\left(m/s\right)$, $\:{x}_{i}$ and $\:{x}_{j}$ represents the spatial coordinate in $\:i$ and $\:j$ direction $\:\left(m\right)$, $\:\mu\:$ represents the dynamic viscosity of fluid $\:\left(Pa .\:s\right)$, $\:\stackrel{-}{{u}_{i}^{{\prime\:}}{u}_{j}^{{\prime\:}}}$ represents the mean turbulent velocity fluctuations $\:\left({m}^{2}/{s}^{2}\right)$ and $\:{S}_{i}$ representing the source term in the momentum equation $\:\left(N/{m}^{3}\right)$.

The $\:SST\:k-\omega\:$ turbulence model is used to capture the air flow turbulence inside the cold storage^13^. The Reynolds stress term in Eq. (2) is simplified according to the Boussinesq hypothesis^27^ as shown in Eqs. (3),

3 $$\:{\rho\:}_{a}\stackrel{-}{{u}_{i}^{{\prime\:}}{u}_{j}^{{\prime\:}}}=-{\mu\:}_{t}\left(\frac{\partial\:{\stackrel{-}{u}}_{i}}{\partial\:{x}_{j}}+\frac{\partial\:{\stackrel{-}{u}}_{j}}{\partial\:{x}_{i}}\right)+\frac{2}{3}{\rho\:}_{a}k{\delta\:}_{ij\:}$$

where $\:{\mu\:}_{t}$ represents turbulent viscosity $\:\left(Pa .\:s\right)$, $\:k$ represents the turbulent kinetic energy $\:\left({m}^{2}/{s}^{2}\right)$ and $\:{\delta\:}_{ij}$ is the Kronecker delta.

The expression for turbulent viscosity $\:\left({\mu\:}_{t}\right)$ in $\:k-\omega\:$ and $\:k-\epsilon\:$ models is shown in Eqs. (4) and (5),

4 $$\:{\mu\:}_{t}={\rho\:}_{a}\frac{k}{\omega\:}$$

5 $$\:{\mu\:}_{t}={\rho\:}_{a}{C}_{\mu\:}\frac{{k}^{2}}{\epsilon\:}$$

where $\:\omega\:$ is the Specific dissipation rate $\:\left(1/s\right)$ and $\:\epsilon\:$ is the dissipation rate of turbulent kinetic energy $\:\left({m}^{2}/{s}^{3}\right)$.

The apple filled crates are modeled as porous medium and is formulated using the Darcy-Forchheimer^28^ equation as shown in Eq. (6). This term is incorporated along with the momentum Eq. (1) as a source term.

6 $$\:{S}_{i}=-\left(\frac{\mu\:}{{C}_{1}}{u}_{i}+\frac{1}{2}{C}_{2}{\rho\:}_{a}\left|u\right|{u}_{i}\right)$$

where $\:{C}_{1}$ is the Darcy permeability (Viscous resistance) $\:\left(1/{m}^{2}\right)$ and $\:{C}_{2}$ is the Forchheimer drag coefficient $\:\left(1/m\right)$.

The energy equation used for modeling the conservation of energy^29^ is shown in Eqs. (7),

7 $$\:\frac{\partial\:{\stackrel{-}{T}}_{a}}{\partial\:t}+{\stackrel{-}{u}}_{i}\left(\frac{\partial\:{\stackrel{-}{T}}_{a}}{\partial\:{x}_{i}}+\frac{1}{{\rho\:}_{a}{C}_{pa}}\frac{\partial\:\stackrel{-}{p}}{\partial\:{x}_{i}}\right)=\frac{\partial\:}{\partial\:{x}_{i}}\left(\frac{{k}_{eff}}{{\rho\:}_{a}{C}_{pa}}\frac{\partial\:{\stackrel{-}{T}}_{a}}{\partial\:{x}_{i}}\right)+{S}_{resp}+{S}_{ingress}$$

where $\:{\stackrel{-}{T}}_{a}$ represents the average fluid temperature $\:\left(K\right)$, $\:{C}_{pa}$ is the fluid specific heat capacity at constant pressure $\:\left(J/kg .\:K\right)$, $\:{k}_{eff}$ is the effective thermal conductivity $\:\left(W/m .\:K\right)$, $\:{S}_{resp}$ and $\:{S}_{ingress}$ represents the additional energy source term due to apples respiration heat load and heat ingress into the cold storage.

The respiration heat generated by the apple^30^ is calculated using the Eq. (8), where the variables $\:f$ and $\:g$ are the respiration coefficients, $\:\gamma\:$ is the porosity of the apple crates, $\:{\rho\:}_{s}$ is the density of apple $\:\left(kg/{m}^{3}\right)$ and $\:{T}_{s}$ is the temperature of the apple crate $\:\left(K\right)$.

8 $$\:{S}_{resp}=\left(1-\gamma\:\right){\rho\:}_{s}\times\:\frac{10.7}{3600}\times\:f{\left[\frac{9{T}_{s}}{5}+32\right]}^{g}$$

9 $$\:f=5.6871\times\:{10}^{-4}$$

10 $$\:g=2.5977$$

### Performance metrics

11 $$\:TD=\frac{1}{\sigma\:\sqrt{2\pi\:}}{e}^{-\frac{1}{2}{\left(\frac{{T}_{s}-\mu\:}{\sigma\:}\right)}^{2}}$$

Temperature density (TD) parameter shows how the temperature of the crates is distributed at a particular time. This data representation is useful to identify the mean temperature of all the crates, $\:\mu\:$ along with their standard deviation, $\:\sigma\:$ how they are spread out from the mean crate temperature. Equation (11) gives the formula for calculating the TD, where $\:\mu\:$ represents the mean of the average value of all the crate temperatures. $\:\sigma\:$ represents the standard deviation which measures the spread or dispersion of the crate temperature data.

12 $$\:{Y}_{i}=\frac{{T}_{s,t}-{T}_{a}}{{T}_{s,in}-{T}_{a}}$$

13 $$\:\text{T}\text{H}=max\left|{Y}_{i}-\frac{1}{n}\sum\:_{i=1}^{n}{Y}_{i}\right|-min\left|{Y}_{i}-\frac{1}{n}\sum\:_{i=1}^{n}{Y}_{i}\right|$$

The thermal heterogeneity is a parameter that is used to gauge the overall temperature gradient within the cold storage^31^. The dimensionless temperature parameter $\:\left({Y}_{i}\right)$, is a form of temperature that has been normalized by dividing it by a reference temperature. The dimensionless temperature parameter is the ratio of difference between the instantaneous apple filled crate temperature and the ambient temperature to the difference between the initial apple filled crate temperature and the ambient temperature. This helps easier comparison and analysis across different systems and scales. The thermal heterogeneity on the other hand, measures the difference between the maximum deviation of $\:{Y}_{i}$ from the mean dimensionless temperature to the minimum deviation of $\:{Y}_{i}$ from the mean dimensionless temperature. The thermal heterogeneity, TH can be determined using the Eqs. (12) and (13), where $\:{T}_{s,t}$ is the apple filled crate temperature at time $\:t$ $\:\left(K\right)$, while $\:{T}_{s,in}$ is the initial temperature of the apple filled crate $\:\left(K\right)$ and $\:n$ is the number of apple filled crates.

14 $$\:\epsilon\:=\frac{{T}_{inlet}-{T}_{return}}{{T}_{a}-{T}_{return}}$$

15 $$\:CEPK=\frac{Compressor\:energy\:consumption\:\left(kWh\right)}{Temperature\:reduction\:\left(K\right)}$$

The cooling effectiveness $\:\left(\epsilon\:\right)$ for each configuration, defined by Eq. (14), compares the actual heat transfer to the maximum possible heat transfer. This parameter evaluates how effectively the apple-filled crates are cooled under similar boundary conditions. Here, $\:{T}_{inlet}$​ refers to the temperature of air entering the cold storage from the cooling unit $\:\left(K\right)$, $\:{T}_{return}$​ denotes the air temperature after cooling the apple-filled crates $\:\left(K\right)$. The parameter Compressor energy per Kelvin (CEPK) normalizes the compressor energy consumed for cooling the crates in each of the pallet configurations and normalizes it to the range of crate temperature reduction. The CEPK is calculated using the Eq. (15).

### Validation

**Fig. 3:**
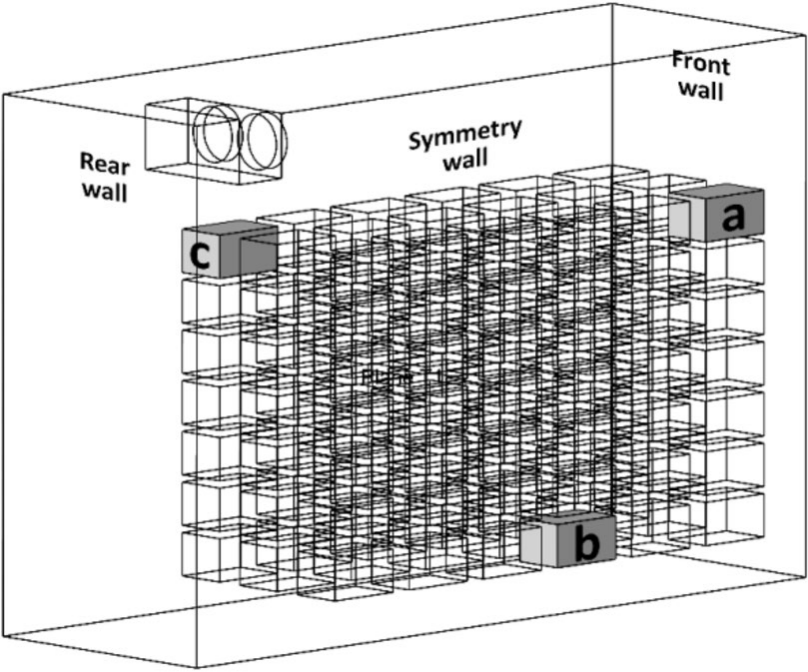
Position of crates considered for validation.

**Table 1:** RMSE and NRMSE values for crates considered for validation.

Crate Position	RMSE (°C)	NRMSE (%)
a	1.135	4.57
b	0.712	2.88
c	0.817	3.87

The modelled cold storage was simulated in ANSYS Fluent v18^32^ and validated against experimental data provided by Bishnoi & Aharwal, (2020)^26^. The position of the crates for which temperature data were validated is shown in Fig. 3. Table 1 shows the RMSE (Root mean square error) and NRMSE (normalized root mean square error) between the simulated and experimental data for crates positioned at a, b and c are found to be 4.57%, 2.88% and 3.87% respectively and are reasonably low. Thus, the model is considered validated.

## Results and discussion

**Fig. 4:**
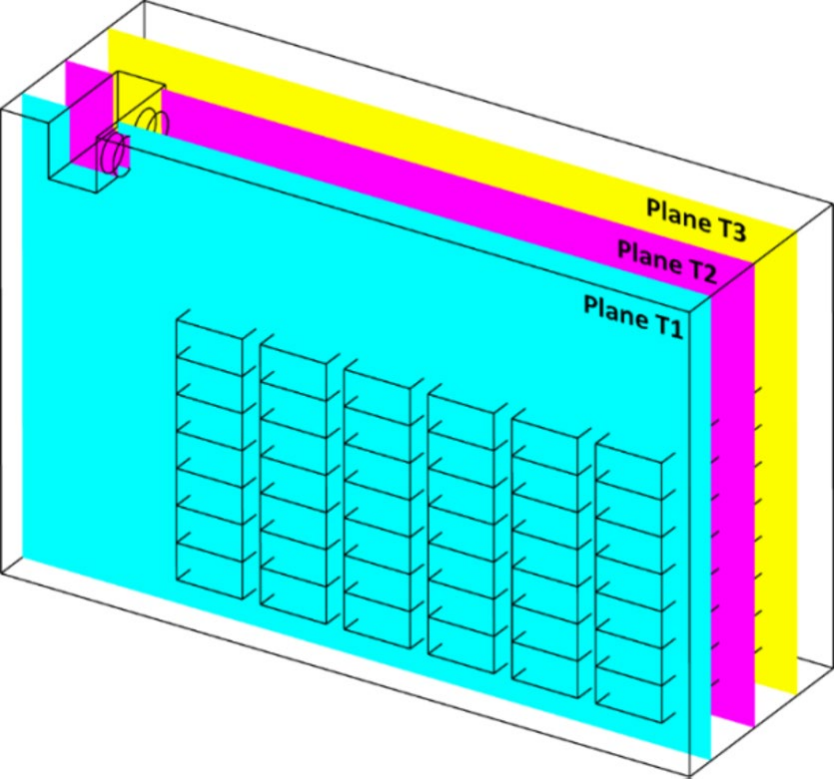
Reference positions for plane T1, T2 and T3.

For monitoring the temperature within the cold storage, three reference planes (T1, T2 and T3) are employed along which the temperature contours are depicted in Fig. 4.

### Temperature distribution

**Fig. 5:**
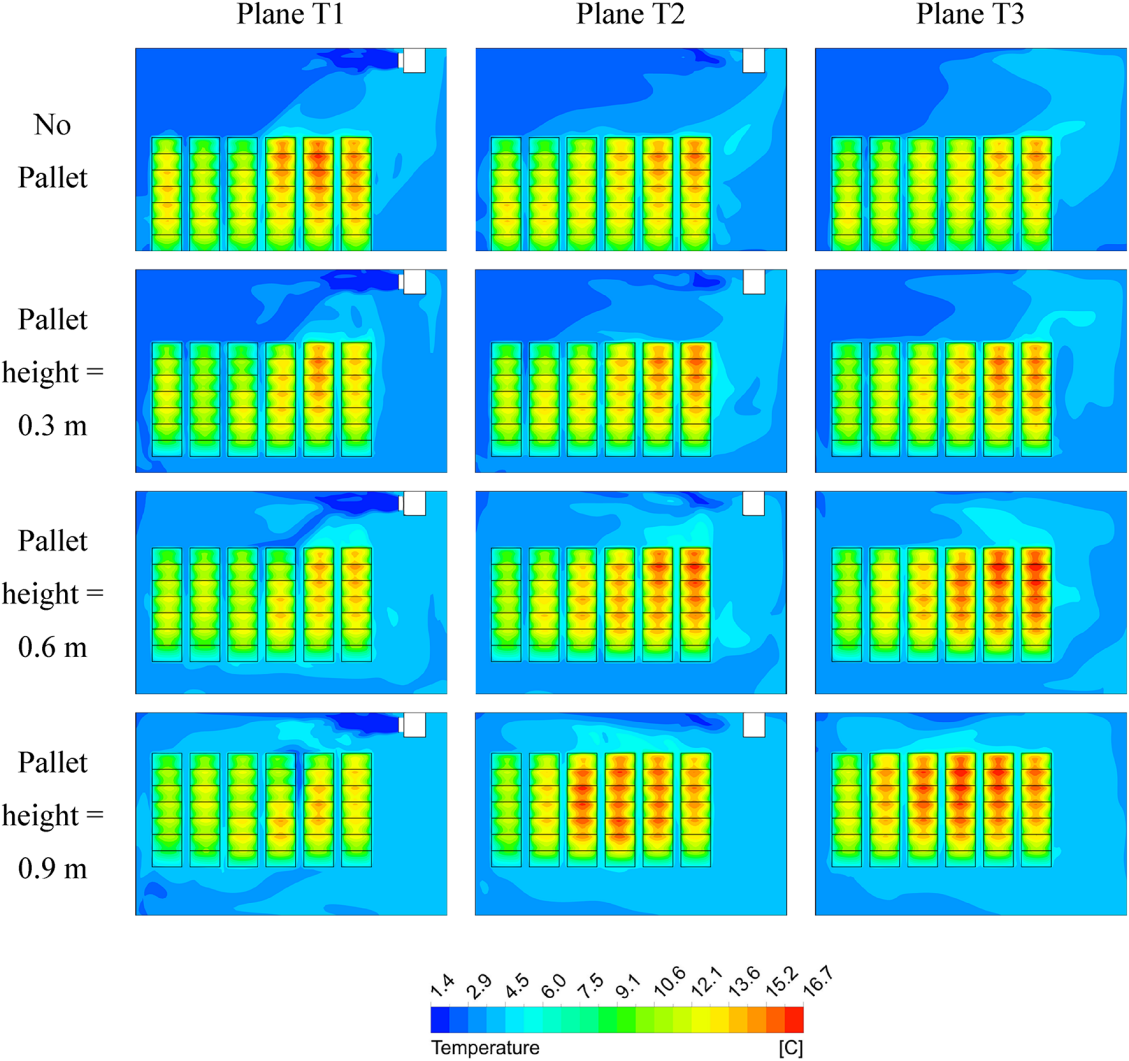
Temperature distribution inside cold storage at 20 h of cooling time.

**Fig. 6:**
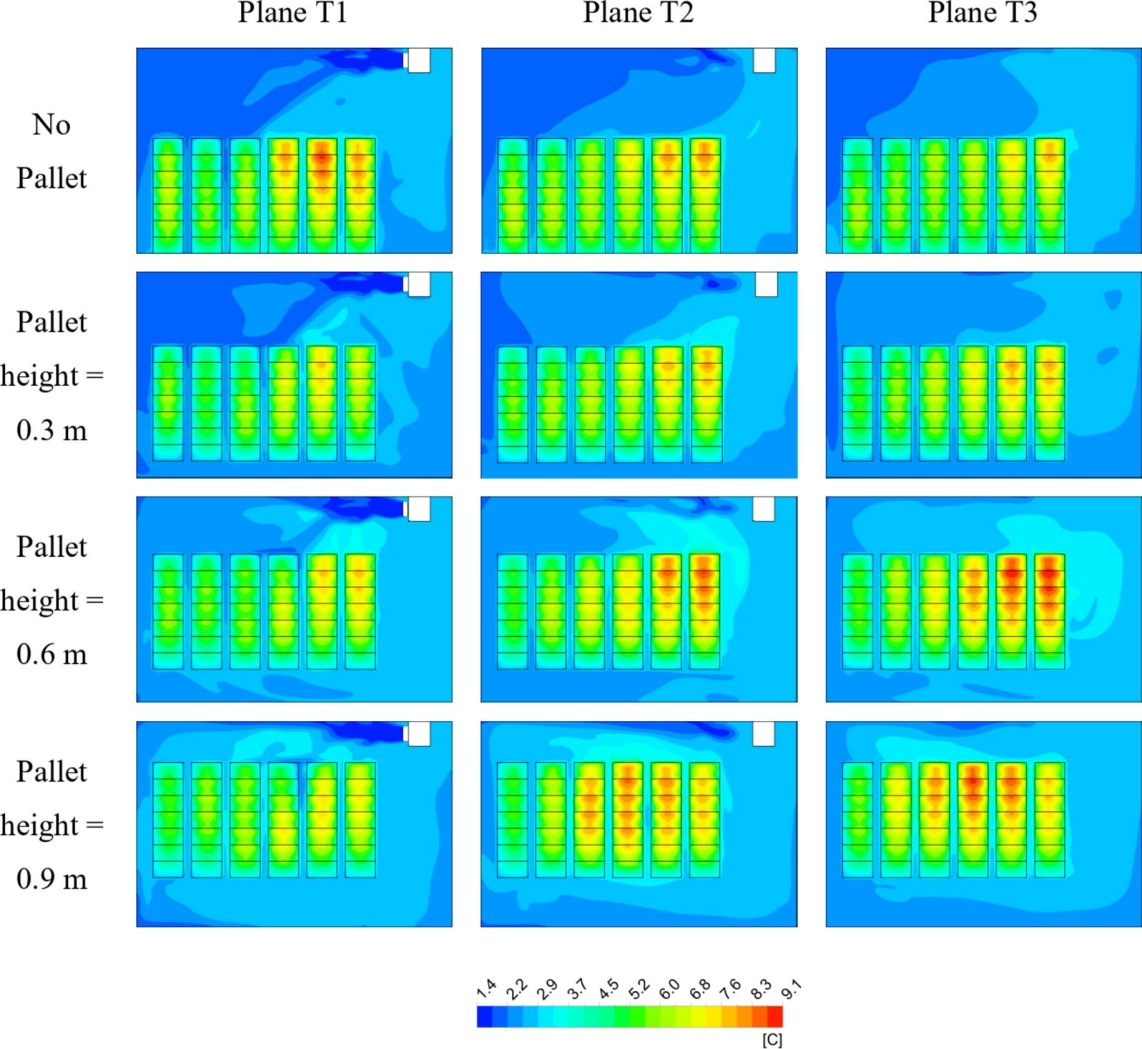
Temperature distribution inside cold storage at 40 h of cooling time.

The temperature distribution diagram is an important parameter which determines the temperature of the crates at different locations inside the cold storage. Different pallet sizes are observed to influence the air flow inside the cold storage within the crates. This gives rise to uneven cooling rates resulting in hot and cold spots arising within the control volume. Uneven cooling inside the cold storage can result in food spoilage and chilling injury. Figures 5 and 6 shows the temperature distribution inside the cold storage at 20 h and 40 h of cooling time, respectively for various pallet heights investigated. From Figs. 5 and 6 it is evident that temperature is unevenly distributed inside the cold storage for pallet height 0.6 m and 0.9 m which attain a maximum temperature of 16.69 °C and 16.65 °C at 20 h of cooling time in T2 and T3 planes. The high temperature zones are visualized in Plane T2 and T3 which comprise 2/3rd of the crates stored. The pattern is similar in 40 h of cooling time also, whereby the highest temperature of 9.11 °C is achieved at 0.6 m and a high temperature of 8.87 °C is achieved at 0.9 m. The impact of pallets is evident when comparing the No pallet configuration with setups that include pallets. At both 20 and 40 h of cooling, crates in Plane T1 experience elevated temperatures in the No pallet configuration. This is due to the absence of a structured airflow path, preventing cold air from reaching the crates located directly beneath the cooling unit. Consequently, inefficient cooling is observed. This inefficiency is reflected by the higher fluid temperatures beneath the cooling units in both figures. Additionally, at pallet heights of 0.6 m and 0.9 m, crates in Plane T3, situated below the cooling unit, show elevated temperatures compared to other crates. This temperature increase results from the larger pallet heights, which allow the cold air to bypass the crates rather than being directed into the spacing between the crates. The 0.3 m pallet height, however, seems to offer an optimal balance between obstructing and facilitating airflow, as it exhibits fewer hotspots relative to other pallet heights for the same cooling duration.

**Fig. 7:**
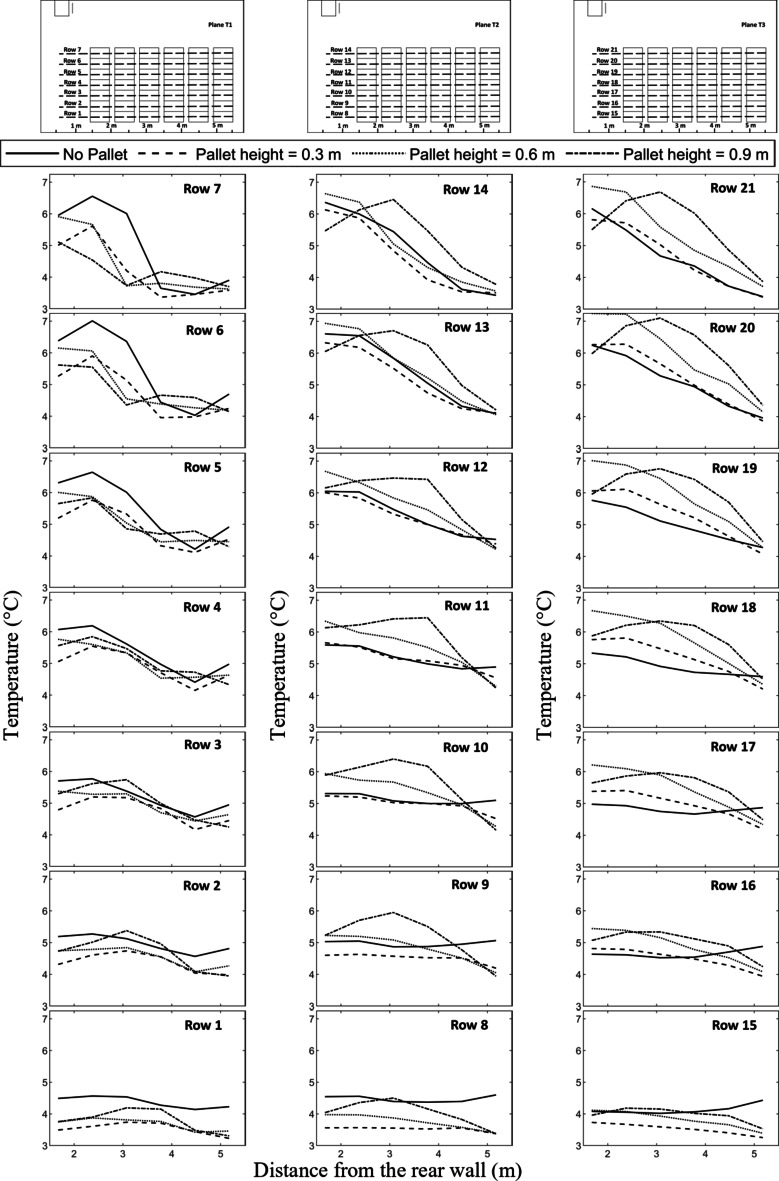
Variation of temperature along the lengthwise direction of the cold storage.

 Figure 7 provides a comprehensive outlook on the average temperature of crates along the lengthwise direction of cold storage. The horizontal axis represents the distance of the crate from the rear wall, while the vertical axis represents the average temperature of the crate at that position. The graph compares the average temperature of the crates on all four different configurations. To represent the average temperature of all the crates and to make a better comparison, separate graphs showing various rows of the crates are plotted. Row numbers are allotted based on the reference plane and position along the Y direction, specified in Fig. 7. From the plots, it can be observed that crates placed at the lower portion (Row 1, Row 8, and Row 15 cool faster than higher ones in all configurations.

It is also observed that the temperature gradient is lowest in Plane T1 of all configurations and the gradient increases in T2 and T3 reference planes. The temperature gradient between the crates within a single row is higher in case of the crates located at the top position (Row 7, Row 14, and Row 21) and the same gradually reduces as it moves to the lower position. This can be visualized as the temperature slope in the plots for crates located at higher positions are larger than the ones located in the lower position. From Fig. 7, it can be concluded that the crates near the cooling unit remain at a higher temperature than any other location. This is due to uneven air flow distribution inside the cold storage. It can be observed that in “No pallet” configuration, the air flow is highly obstructed which reduces the vertical movement of cold air for cooling the crates near the cooling unit. The opposite effect can be observed in the temperature contours for pallet heights 0.6 m and above. In these configurations, air flow is unobstructed, reducing the contact of chilled air with the crates located near the cooling unit. Pallet height of 0.3 m is adjusted better as it doesn’t fully obstruct the air flow completely thus allowing for the required amount of chilled air to rise vertically and cool down the crate near the cooling unit. By analyzing the performance metrics of the crate temperatures in all four configurations, the optimum pallet height can be determined.

### Time-Dependent temperature variation

**Fig. 8:**
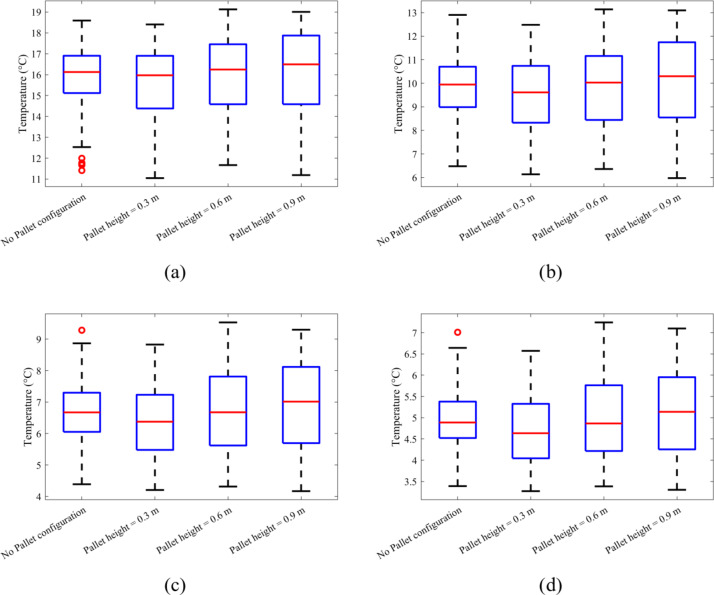
Box Whisker plot for temperature distribution over time (**a**) 10 h, (**b**) 20 h, (**c**) 30 h and (**d**) 40 h.

Figure 8 provides a detailed representation of the temperature distribution for crates at 10, 20, 30, and 40 h of cooling, using box whisker plots. The box plot represents the median temperature in its middle with a line, and top and bottom box edges representing the 25th and 75th percentile. The whiskers extend to cover data points up to 1.5 times the interquartile range, while the box height illustrates the interquartile range. Outliers are indicated by circles which essentially represent crates with temperatures outside the whisker range, if any. A larger box height thus signifies a greater number of crates within the interquartile range, indicating more consistent cooling. At 10 h of cooling time, the average crate temperature in all configurations is found between 16 and 16.5 °C which indicates a better and uniform cooling across all configurations. It can also be identified that at 10 h, only the No pallet configuration consists of around five outliers which can be attributed to the poor air circulation near the crates located at the bottom due to the absence of pallets. After 10 h of cooling, the 0.9 m pallet height shows a box height of 3.29 °C which is significantly higher than the 1.8 °C observed in the No pallet configuration. This suggests that increased pallet height has a detrimental effect on crate cooling. It can also be noted that the lowest mean crate temperature is achieved in case of 0.3 m pallet height and box plot height is 16 °C.

Across the 20, 30, and 40-hour cooling periods, the same pattern emerged, with the 0.3 m pallet showing the lowest mean temperatures of 9.6 °C, 6.4 °C, and 4.7 °C. The data revealed that as the cooling time increased, the temperature range narrowed further to 0.68 °C, 0.64 °C, and 0.5 °C, demonstrating improved uniform cooling over time. Notably, one outlier remained in the No Pallet configuration even at 30-hour and 40-hour intervals, indicating severely restricted air flow in this arrangement in this configuration. After 40 h, the 0.3 m pallet configuration is shown to have the lowest mean temperature, lowest 25th and 75th percentiles, and the smallest maximum crate temperature.

### Performance metrics

**Fig. 9:**
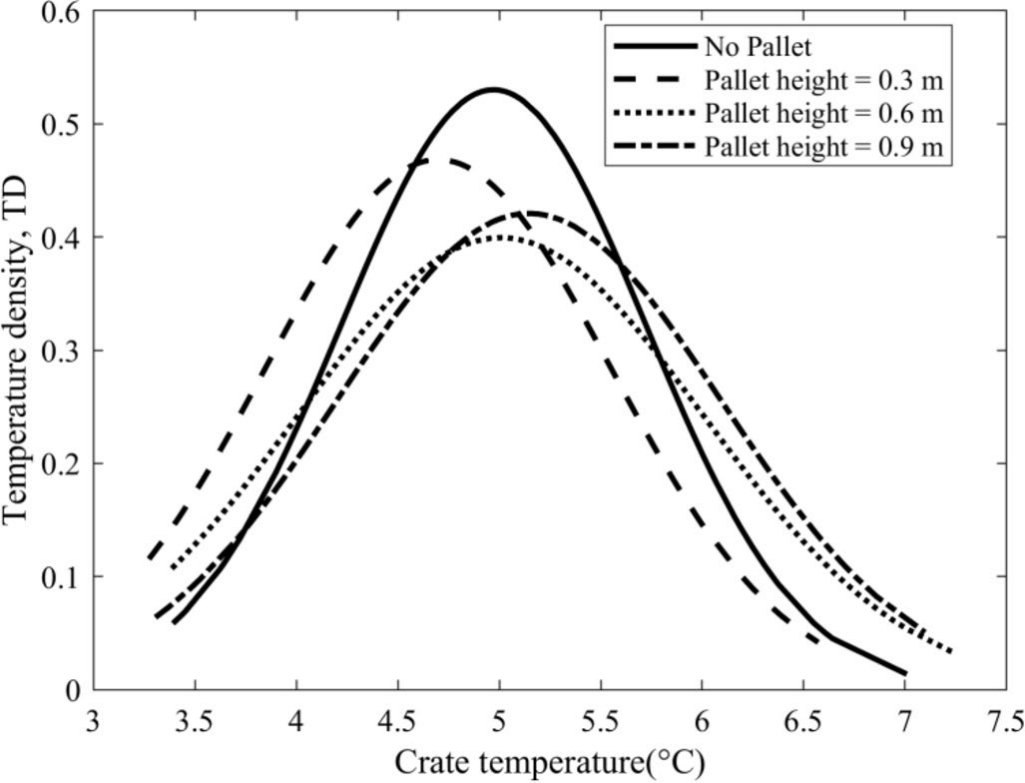
Temperature Density plot at 40 h of cooling.

**Table 2:** Temperature density parameters at 40 h of cooling.

Pallet configuration	Mean (°C)	Standard Deviation	Skewness	Maximum temperature (°C)	Minimum temperature (°C)
No Pallet	4.97	0.75	0.43	7	3.4
0.3 m Pallet	4.7	0.85	0.22	6.57	3.27
0.6 m Pallet	5	1	0.34	7.24	3.38
0.9 m Pallet	5.15	0.95	0.02	7.1	3.31

The temperature density plot exhibits a bell-shaped curve, with the mean crate temperature positioned at the peak, and the width of the curve signifying the standard deviation of crate temperatures in each configuration (Fig. 9). A narrower curve suggests more uniform crate temperatures in the cold storage, whereas a wider curve reflects greater temperature variation. The No pallet configuration achieved the highest maximum temperature density of 0.53 at a crate temperature of 4.97 °C, indicating that most crates were cooled around this mean temperature. Conversely, the lowest temperature density was observed in the 0.6 m pallet configuration, with a value of 0.4 at a crate temperature of 5.01 °C. The 0.3 m and 0.9 m pallet heights exhibited temperature densities of 0.47 at 4.7 °C and 0.42 at 5.15 °C, respectively. This data indicates that the crate temperature uniformity follows the order of No pallet, 0.3 m, 0.9 m, and 0.6 m pallet heights. As seen in Fig. 9, the bell curves are not perfectly symmetric, the skewness of the curves provides more insight into the process. The skewness values as mentioned in Table 2 for all configurations are positive and lean towards the right implying the cooling process of the crates. While the No pallet configuration yielded the highest temperature density, the 0.3 m pallet height achieved the lowest average crate temperature, which is preferable.

**Fig. 10:**
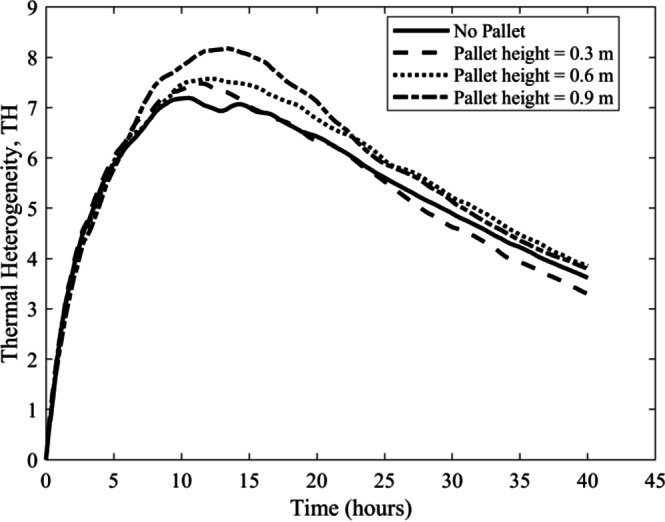
Thermal heterogeneity measured with time.

The thermal heterogeneity (TH) parameter provides a measure of temperature uniformity within a cold storage system for a given configuration at a specific time. Lower TH values indicate more homogeneous cooling inside the cold storage. Figure 10 presents the TH versus time plot which offers insight for both the maximum and minimum crate temperatures. The TH values for all configurations exhibit a similar trend, starting with an initial increase followed by a gradual decrease. This behavior can be explained by the fact that at the beginning, the heat rejected by the crates exceeds the cooling capacity, but over time, the cooling effect becomes more dominant. The highest TH value of 8.18 was observed for the 0.9 m pallet height at 13.33 h, followed by the 0.6 m pallet height (TH = 7.57 at 11.67 h), the 0.3 m pallet height (TH = 7.48 at 11.38 h), and the No Pallet configuration, which showed the lowest peak value of 7.19 at 10.55 h. Although the No Pallet configuration exhibited the lowest maximum TH value initially, the 0.3 m pallet setup showed a steeper decline in TH values after 21.1 h, eventually becoming lowest. After 40 h of cooling, it is clear from the plot that crates stored with a 0.3 m pallet height achieved the lowest TH value of 3.29, outperforming other configurations by 8.79% (No pallet), 14.42% (0.6 m pallet height) and 13.03% (0.9 m pallet height), respectively.

**Fig. 11:**
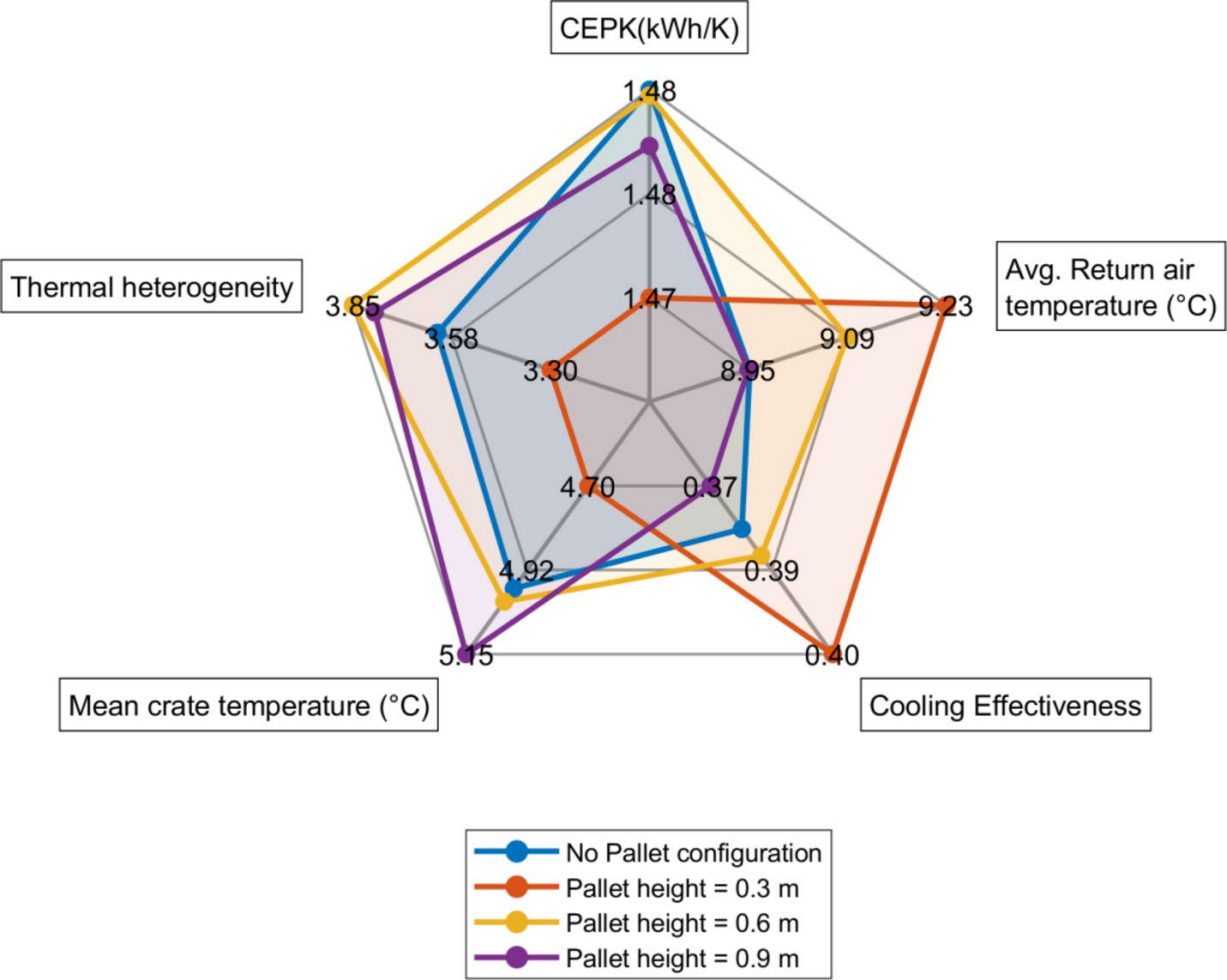
Radar Plot comparing the performance metrics.

Figure 11 presents a radar plot illustrating performance metrics, including CEPK (kWh/K), average return air temperature (°C), cooling effectiveness, mean crate temperature (°C), and thermal heterogeneity. Among the tested configurations, the 0.3 m pallet height is found to have the lowest CEPK of 1.474 kWh/K, followed by the 0.9 m, 0.6 m, and no-pallet configurations, which consumed 1.479 kWh/K, 1.481 kWh/K, and 1.482 kWh/K, respectively – representing increases of 0.36%, 0.49%, and 0.5% compared to the lowest value. The average return air temperatures for the configurations: No pallet, 0.3 m, 0.6 m, and 0.9 m configurations were recorded as 8.95 °C, 9.22 °C, 9.08 °C, and 8.95 °C, respectively. The increase in return air temperature for the 0.3 m configuration indicates improved airflow distribution within the cold storage, resulting in faster cooling of the crates. This is supported by cooling effectiveness data, where the 0.3 m pallet configuration achieved the highest effectiveness of 0.4, compared to 0.383, 0.38, and 0.374 for the 0.6 m, 0.9 m, and no-pallet configurations, respectively. These findings suggest that the 0.3 m pallet height enables the most efficient cooling among the tested configurations. The findings of this study are based on a specific cold storage configuration and may vary depending on storage geometry and airflow patterns.

## Conclusion

Food wastage can aggravate due to uneven cooling of the stored products caused by poor air flow distribution inside the cold storage. In this research, the effect of the pallet height on the cooling rate, measured in terms of crate temperature is studied. A cold storage model based on literature was modeled and validated using CFD. Four different pallet configurations were considered based on the validated cold storage model. Crates stacked with no pallets, crates stacked on pallet heights 0.3 m, 0.6 m, and 0.9 m. The model of the cold storage was validated against experimental data and the different pallet configurations were simulated. The temperature distribution along three reference planes were studied. The temperature variation across each row of crates was analyzed for different pallet height configurations to determine how pallet height affects the temperature profile of crates along the vertical axis. In addition to temperature distribution, statistical tools such as box-whisker plots—displaying the mean temperature, 25th and 75th percentiles, whiskers, and outlier data—were employed, along with standard deviation analysis, temperature density plots, and thermal heterogeneity assessments. These statistical analyses were used to examine the effect of pallet height on the cooling performance of apple crates stored in cold storage. While the CFD model effectively reduces the cost and complexity of real-life experimentation, it relies on certain assumptions, such as idealized boundary conditions, and simplified turbulence model. These simplifications, while being necessary for computational efficiency, may introduce deviations when compared to actual cold storage operations. Future studies incorporating real-time variations and advanced turbulence models could further enhance the model’s accuracy. The results of this study provide insights into how varying pallet heights influence cooling efficiency. The key findings drawn from the analysis are presented below.

- Temperature contours were analyzed for four configurations at both 20 and 40 h of cooling time. It was observed that crates nearest and beneath the cooling units consistently exhibited higher temperatures across all configurations, a result of typical airflow patterns within the cold storage. Crates placed on 0.3 m pallets showed fewer hot spots at both cooling durations of 20 and 40 h, achieving the lowest average crate temperature of 8.14 °C. In contrast, the no pallet configuration and pallets with heights of 0.6 m and 0.9 m exhibited temperatures 8.47%, 11.9%, and 8.9% higher, respectively.
- A row-wise analysis was conducted to further explore the effect of pallet height on crate temperature. It was identified that crates positioned in the top rows (rows 6, 7, 13, 14, 20, 21, refer to Fig. 7) experienced a temperature difference of up to 74.2% compared to other crates in the lower row. This is attributed to the typical airflow distribution within the storage. The smallest temperature difference (48.7%) in the top rows was observed for the 0.3 m pallet height configuration, with temperature differences diminishing further down the stacks. In all configurations, crates below the cooling units exhibited the highest temperatures.
- A box whisker plot was used to analyze various cooling, revealing that mean crate temperatures in all configurations remained within ± 0.1 °C at 10 h but increased to ± 0.25 °C at 40 h. The 0.3 m pallet configuration achieved the lowest mean temperature.
- Temperature density (TD) plot, considering both mean and standard deviation values, were also analyzed. The No pallet configuration showed the highest TD value of 0.53, while the 0.3 m, 0.6 m, and 0.9 m pallet heights displayed TD values 11.3%, 24.5%, and 20.7% lower, respectively. Although No pallet configuration achieved the highest TD value, the dominance of lower mean crate temperatures in ensuring optimal storage conditions supports the conclusion that the 0.3 m pallet height is the most effective.
- Thermal heterogeneity (TH), calculated for all configurations revealed that pallet height of 0.3 m obtained the lowest TH after 40 h of cooling time. The 0.6 m pallet height achieved 14.42% highest TH while No pallet and pallet height of 0.9 m achieved 8.79% and 13.03% higher TH respectively. The results are conclusive that the pallet height of 0.3 m height achieved better cooling compared to all other configurations.
- The improved airflow distribution in case of 0.3 m pallet height results in a lower CEPK and a higher average return air temperature. For this configuration, the CEPK determined was 1.474 kWh/K, and the average return air temperature was 9.22 °C, marking the lowest CEPK and highest return air temperature. In contrast, the no-pallet configuration recorded the highest CEPK of 1.482 kWh/K and the lowest average return air temperature of 8.95 °C.
- In terms of cooling effectiveness, the 0.3 m pallet height outperformed other configurations with a value of 0.4. The 0.6 m, 0.9 m, and no-pallet setups showed slightly lower values of 0.383, 0.38, and 0.374, respectively.

As observed, the 0.3 m pallet height configuration enhances airflow distribution and cooling efficiency, making it a viable option for optimizing energy use. The 0.3 m pallet configuration also demonstrates the highest cooling effectiveness in our simulations and its practical implementation looks quite feasible in most of the cold storages with minimal effort.

## Electronic supplementary material

Below is the link to the electronic supplementary material.

Supplementary Material 1

## Data Availability

The data generated or analyzed during the parametric study are included in this published article and its supplementary information files. The CFD analysis data can be obtained from the corresponding author, Leo Daniel Alexander, upon reasonable request.

## List of symbols

${C}_{1}$: Darcy permeability (Viscous resistance) $\left({m}^{-2}\right)$
${C}_{2}$: Forchheimer drag coefficient $\left({m}^{-1}\right)$
$${C}_{p}$$: Sensible heat $$\left(J {kg}^{-1} {K}^{-1}\right)$$
${C}_{\mu }$: Turbulence model constant
$$CR$$: Cooling rate $$\left(K {s}^{-1}\right)$$
$g$: Acceleration due to gravity $\left(m {s}^{-2}\right)$
$$I$$: Turbulence intensity ($$\%$$)
$k$: Thermal conductivity $\left(W {m}^{-1} {K}^{-1}\right)$ / Turbulent kinetic energy $\left(J {kg}^{-1}\right)$
$l$: Turbulent length scale $(m)$
$$p$$: Pressure $$\left(N {m}^{-2}\right)$$
$$S$$: Source term
$$Sc$$: Schmidt number
$$t$$: Time $$\left(s\right)$$
$$T$$: Temperature $$\left(K\right)$$
$$u,U$$: Velocity $$\left(m {s}^{-1}\right)$$
x: Displacement $$\left(m\right)$$

## Greek

$$\gamma$$: Porosity
$${\delta }_{ij}$$: Kronecker delta
$$\mu$$: Dynamic viscosity $$\left(N s {m}^{-2}\right)$$
$${\mu }_{t}$$: Turbulent viscosity $$\left(N s {m}^{-2}\right)$$
$$\rho$$: Density $$\left(kg {m}^{-3}\right)$$
$\omega$: Specific turbulent dissipation rate $\left({s}^{-1}\right)$
$$\tau$$: Cooling time $$\left(s\right)$$

## Sub-script

a: Air
eff: Effective values
e: Energy parameter
fi: Final
i: Vector representing x direction
in: Initial
int: Heat exchange between the apple and the air
j: Vector representing y direction
resp: Respiration heat
s: Solid (apple)
w: Water vapor
